# Clinical Review and Hospital Reports

**Published:** 1842-04-01

**Authors:** 


### Clinical Review.

---

### DUNDEE ROYAL ASYLUM FOR LUNATICS.

### Twentieth and Twenty-first Annual Report.

We shall notice one or two particulars in these Reports.

*Causes of Insanity.*—Hereditary predisposition is found to be the most prominent. There are at present in the house a mother and her daughter, both admitted since last report. In families in which there is an hereditary tendency, though the disease may be latent in all the members, yet in many of them may be observed oddities of manner, and eccentricities, which a very slight cause is often sufficient to develope into insanity. Drunkenness continues, in many instances, to be an exciting cause, though probably not to the extent which many ascribe to it. The reporters have been inclined, in several cases, to think that, instead of being the cause of the disease, it was the consequence of its gradual approach; that the patient might have had recourse to intoxicating liquors to drown the uneasy sensations and troubled thoughts arising from approaching insanity. In other cases, the malady has evidently been kept up, and fits of excitement produced, by a disordered state of the alimentary canal. In such cases, till the bowels are brought into a healthy condition, which it often is very difficult to do, there is little chance of cure; but when once they have been got to act in a healthy manner, the disease often disappears very quickly.

*Consulting Patients' Tastes and Habits.*—If a patient has been accustomed to a suite of apartments, by placing him on a rate of board sufficiently high to provide him with this accommodation, an obstacle to his cure is at once removed, and he feels no degradation from his change of abode; but if he is treated differently, his irritation will be great, and his cure more protracted. One grand object is, to make the lunatic feel little or no difference by the change in regard to the comforts of life.

The reporters always endeavour to humour the whims of the old cases as much as possible. One gentleman, after tearing his linen to pieces, demanded, in a loud tone of voice, muslin shirts. A common cotton shirt was sent to him, and having been requested to say whether that was the kind wanted, he answered in the affirmative. He has worn these shirts for some time in the most satisfactory manner, and taken the greatest care of them.

*Non-Restraint System.*—Our readers are aware that we have all along opposed what we consider as great a piece of insanity as any to be found in the patients themselves—we mean the total abolition of restraint. We believe that this is, after all, impossible, one species of restraint, the manual, being only substituted for another. And at what a cost! In short this is one of the "humbugs" of the day. Let us hear what the reporters say to it.

"The abuses which were prevalent in madhouses in this country many years ago, and which were so fully exposed in several publications, were, when generally known, calculated to arouse the minds of all to the inquiry how far restraint of every kind might be done away with. There are probably some places in England where great improvements in the matter of restraint might still be made; but in the most of those which are well regulated the restraint used is reduced to a mere trifle. Some, we hear, have gone the length of laying it aside in all cases; but a regard to humanity imposes upon us the indispensable necessity of using some means of restraint to prevent the violence of a patient from being fatal either to himself or attendants; and in others to use it as a method of cure to prevent the patient from exhausting his strength by violent muscular exertion during a paroxysm, which, unless restrained, might end in fatal depression.

There are paroxysms at times so violent that no moral influence can have any effect in alleviating them; on the contrary, they are heightened by the mere presence of an attendant. Besides these reasons, there are others which at times render restraint imperative; but as this report is for general, and not professional perusal exclusively, we are obliged to withhold them from the public at large. Restraint may be either active or passive: in the former we have the patients and attendants perpetually struggling; in the latter we have the means of prevention and comparative tranquillity without loss of strength. We admit that an increase in the number of attendants must diminish the number under confinement by mechanical apparatus; but what we contend for is, that if the English lunatics, who are said to be 'non-restrained,' are like the Scotch, when in certain states of excitement, restraint cannot be dispensed with, in all cases, without positive injury. No matter how many attendants could be got, by night as well as by day, to hold with their hands, strive, and attempt to overawe lunatics labouring under furious, or any other kind of mania requiring restraint—from our experience we consider such a system to be neither safe nor proper, and not to be compared to one of a mild and passive nature. It is worthy of remark that there is at least one patient in this asylum who has frequently felt and acknowledged the good effects of temporary restraint, and who, on the approach of the paroxysm cries out lustily for the straps to be applied, to prevent mischief from being committed. Occasionally, all our patients are to be found perfectly free; and the very small number that we have at any time under mechanical confinement, is a proof that we are not advocates for restraint wherever it can be safely dispensed with. Seclusion, regulated according to circumstances, and attended with sufficient restraint to prevent mischief, we hold, in common with almost all who have had much experience in the management of the insane, to be the most effectual and the most humane means of allaying violent paroxysms. It is absolutely necessary also for the sake of the other patients. A lunatic breaking out into a paroxysm among a number of other patients—not to mention the danger to which his violence may expose them—would, unless speedily removed, be the cause of others falling into the same state. From almost all the Reports of other Institutions with which we are favoured, we see that restraint, though used as seldom as possible, is yet considered at times indispensable; and from other information now before us, and to which we will not farther allude at present, we are convinced that what is called 'non-restraint' is a system that must injure the patients of a certain class who are the subjects of it, as well as those in attendance upon them."

The reporters, Dr. Nimmo and Mr. Mackintosh, speak like plain and sensible men.

*Delusions of the Insane.*—The Reporters notice these at some little length. We have, they say, as usual, several patients who fancy themselves to be gods. Some of them admit the divinity of the others—arrogating to themselves, however, the title of the supreme deity, and allowing the others to be only inferior divinities. They have never observed this delusion to occur in females—possibly our holy religion may have some influence in this exemption; and that in countries where many gods, *male* and *female*, are worshipped, there instances may occur of female lunatics suffering under this delusion. There is a very common delusion, branching out into many varieties, of which we have several instances. That in which the patients suppose that their friends, or the medical officers or servants of the Institution, are practising on them for the purpose of keeping them in an Asylum. They have several patients who assert that they are literally drugged with medicine for that purpose, and also to injure their bodily health. They are in excellent bodily health, of course require very little medicine, and get it very seldom. Another patient fancied that the food which was served up to him was principally composed of poisonous reptiles; and at times, in consequence of this delusion, refused all kinds of nourishment, especially fluid, in which he thought that he was more apt to be deceived. Another has taken up the notion that the wine which she is sometimes allowed on account of her health is nothing but the blood of her children, and that it is given her solely for the purpose of prolonging her insanity. No less than four females adopted a similar notion, viz., that the legs of their own children were presented to them under the form of legs of mutton, for the same purpose. They were consequently under the necessity of discontinuing this article of food to them for some time, till returning reason dispelled the delusion.

Another very common form of delusion, of which they have several varieties in the Asylum, is that wherein the patients think they are under the influence of evil spirits, or that incantations are practised on them, or that they hold conversation with imaginary beings invisible to all around, and frequently communicating with them from foreign lands. The superstitious character of many of the lower orders of Scotch would seem to give encouragement to this delusion.

Another, not uncommon, delusion is that in which the unhappy patient imagines that he has committed great crimes and is an object of Divine wrath. A wretched and an obstinate form of insanity. But some patients recover from it notwithstanding.

" The last kind of delusion which we shall at present notice, is that under the influence of which the patient is impelled to the commission of violent crimes. The history of crime, in all ages and countries, abounds with cases of violence and murder committed by persons of insane minds, often on those to whom they were most nearly related, and most warmly attached, under the delusion, in some cases, that they were rendering an acceptable service to their Maker; in others, that they were performing an act of kindness to their victims, in removing them from the miseries and persecutions of the world. The motives to the commission of crime by lunatics are infinitely varied. Those already mentioned are probably among the most common. It would be endless to attempt to enumerate the more singular cases, but we may allude to one case which must be familiar to all as an example of delusion of a very singular kind, impelling the patient to the commission of crime. We allude to the case of Hatfield, who fired at his late Majesty George the Third. He laboured under the delusion that he was destined to save mankind by dying a violent death. Suicide, though a violent death, was his own act, and a criminal one. He could not, therefore, support the character of a saviour of mankind if he took away his own life. He must be put to death by others, and though he could see the incompatibility of the character of a saviour of mankind and a self-murderer, he did not see that the murderer of his neighbour was equally disqualified for his supposed office. But it is not alone lunatics, labouring under such delusions, from whom deeds of violence may be feared. A lunatic is a person whose mind we never can calculate upon continuing long in one state, nor can we anticipate the changes which will be produced in it with the same degree of probability that we can do in the case of a sane mind, when acted upon by similar influences. This is more especially the case with those who have been naturally imbecile. A lunatic, in a state of excitement, is always to be dreaded. Though under no delusion, the excitement itself may render him utterly unconscious of his actions; and in such a state, unless carefully watched or restrained, he may commit violence on himself or others. Persons naturally imbecile, or verging on idiotcy, are well known to be in many cases highly excitable, and subject to the most ungovernable bursts of passion from the most trifling causes; under the influence of which they have committed murder, accompanied with circumstances of the most horrid barbarity. In such cases, though they have been the instruments of depriving a fellow-creature of life, yet they have been the unconscious instruments. We cannot regard them in the light of criminals, for they wanted that which in all cases is essential to crime, —namely, the knowledge that they were doing wrong. Many such accidents might be prevented if the disease were attended to at its commencement, and the unfortunate sufferers placed under restraint; and we have no doubt that institutions such as our own have been the means of preventing a great many such cases from occurring. We have also little doubt that a great many of our patients, had they been left at liberty, might, at some time or other, have committed crimes of a like nature.

It cannot, therefore, be too strongly or too frequently inculcated on the minds of all, that early restraint is not only the most effectual means of relieving the unfortunate lunatic, but also the greatest safeguard of the public from their unconscious violence, or from the no less fatal consequences of their delusions. We cannot drop this subject without expressing our regret, that, in many parts of the country, numbers of these unfortunate beings are allowed to wander about, subject often to the most unfeeling jests and the most brutal usage, and almost entirely dependent on casual charity for a most miserable existence."

---

### GENERAL HOSPITAL AT PICOLATA.

### Report of a Case of Axillary Aneurism. By C. M'Dougall, M.D. U.S.A.*

John Kane, æt. 24, was shot by the Indians, and wounded in two places; one ball striking midway between the 8th dorsal vertebra and the angle of its rib, appeared in front underneath the skin; the other entering about two inches above the lower angle of the scapula, divided the axillary artery about its middle; there was great hæmorrhage, and he was brought to the hospital in a state of insensibility. After the system had rallied, a ball was extracted from under the skin of the left side, the integuments around the anterior upper wound were found much distended, having a dark livid appearance. At the end of a fortnight, the effused blood and swelling had disappeared, the external wounds were healthy, and the patient was considered out of danger; he continued to improve until the end of the fourth week, when a small pulsatory tumor appeared over the axillary artery, and directly under the external wound; this continued to increase, and, on the 10th day, extended above to the clavicle, pushing that bone out of its natural position, and anteriorly, putting the pectoral muscles so much on the stretch, as to threaten momentarily to give way; pulse at the wrist almost imperceptible; arm insensible to the touch, and motionless.

Apprehending an immediate rupture of the tumor, it was determined to tie the subclavian artery above the clavicle, which was done on the following day; all pulsation at the wrist, and in the tumor, immediately ceased. From this time, the patient went on favourably, the tumor diminishing rapidly, the arm regained its natural warmth, and was fast recovering its sensibility, until the sixth day after the operation, when an alarming hæmorrage took place, which was at last arrested by syncope; on the following day, the bleeding was renewed, and at 12 P. M. he expired.

*Sectio Cadaveris.*—The pectoralis major was much attenuated; the blood had been forced through its fibres, as through a sieve, and in some places appeared incorporated with its substance. The same was the case with the pectoralis

---

* The Maryland Medical and Surgical Journal.

minor. Underneath these muscles, an immense mass of coagulated blood, partially organized was discovered. The axillary artery was found completely divided, and the ends retracted about half an inch. Not the slightest adhesion had taken place, the mouths being as patulous as if just cut; veins and nerves uninjured. The subclavian artery was found ulcerated where the ligature had been applied, and presented a scolloped uneven edge at both orifices. No trace of adhesive inflammation in it could be discovered.

---

### Clinical Remarks of M. Velpeau on Abscess of the Iliac Region.*

*Causes of Iliac Abscess.*—They may exist in the hard or soft parts. Inflammation of the muscles, of the cellular tissue, of the peritoneum, or of the bones of this region, may act as an exciting cause, as well as inflammation of distant parts. Thus, various diseases of the kidneys, of the cœcum, or sigmoid flexure of the colon, may give rise to the presence of purulent collections in the iliac fossa; they may also depend on perforation, scirrhus or cancer of the intestines, diseases of the chord and testicle, hernia and the operations for its relief, diseases of the bladder, or prostate gland, the urethra, &c. Iliac abscess may depend on disease of the groin, or thigh; thus inflammation of the *bursa mucosa* of the psoas and iliacus muscles, or of the hip-joint, is a frequent cause of iliac abscess. Disease of the female genital organs is, likewise, a very frequent cause of this affection. Diseases of the ovary, for example, may give rise to two species of iliac abscess. When the serous lining of the ovary is inflamed, the pus which descends to the iliac fossa is contained in the cavity of the peritoneum; but if the substance of the ovary be inflamed, the abscess is then seated outside the cavity.

Diseases of the womb are very often an exciting cause of abscess in the iliac region; this we can readily understand. In cases of metritis, the inflammation easily extends to the cellular tissue of the broad ligaments, and with them to the iliac fossa.

Disease of the bone is a frequent cause. Buboes may give rise to it. There is in the hospital a young student in medicine, who had been affected with bubo; the tumor suppurated, and the inflammation extending from the glands in the groin to those in the iliac fossa, the consequence has been the formation of abscess in the latter part.

*Seat of the Abscess.*—The abscess may be situate first, in the parietes of the abdomen covering the iliac region; second, in the iliac fossa underneath the peritoneum; and third, in the peritoneal cavity.

*Abscess in the Abdominal Walls.*—Inflammation of the inguinal glands is frequently the cause of this species of iliac abscess; it may also depend on inflammation of the chord, hernia, or on operations performed in this region. Abscesses in the abdominal wall of the iliac region are of three sorts; first, they may be seated between the integument and the fascia of the external oblique muscle; in this case they may be extensively diffused. Diseases of the urethra may occasion this diffused inflammation—for example, ulceration at the spongy or bulbous portion of the canal.

Second, the abscess may be situate in the inguinal canal.

Third, it may have its seat between the peritoneum and the muscular wall of

---

* Prov. Med. and Surg. Journ. Feb. 12, 1842.

the abdomen, behind. These are important distinctions, because the treatment must necessarily vary according to the seat of the abscess.

*Abscess in the Iliac Fossa.*—The following observations are extremely good, though rather long. "These abscesses always depend on inflammation of the peritoneum, or genital organs, and present some varieties with respect to their seat, which you should be acquainted with. In some cases the abscess is seated beneath the peritoneum; sometimes under the fascia iliaca; sometimes in the peritoneal cavity. When the pus collects under the peritoneum, it extends rapidly towards the flank or in the walls of the abdomen, extending even to the inguinal canal. These abscesses may depend on diseases of the kidney, caries of the spine, ribs, or pelvis, and diseases of the womb or genital organs. The third species is that in which the abscess is situate under the fascia iliaca. This layer, arising from the fibrous band which envelops the origin of the psoas muscle, becomes gradually thicker as it descends to the iliac fossa; here it encloses the anterior circumflex artery, and joins the fascia transversalis; over the fleshy part of the psoas muscle it is thin, but it thickens over the tendinous part, and passing downwards joins Poupart's ligament. The iliac fascia thus forms the anterior half of the iliac canal, which contains the psoas and iliacus muscles, and terminates inferiorly in the thigh. The superior aperture of the iliac canal is limited behind by the ilio-lumbar ligament with the transverse process and body of the last lumbar vertebra. The fibrous sheath of the psoas connects the fascia iliaca to the ligament of the diaphragm, and hence we have a long trajet continuous from the diaphragm to the lesser trochanter. This brief description will help to explain to you the symptoms arising from the presence of pus in the iliac canal. When the pus comes from inflammation of the cellular tissue of the iliac fossa, or some more distant part, it must follow a certain course; if it descends between the peritoneum and fascia iliaca, it will come out by the crural or inguinal canal, and the abscess will be superficial. In cases of psoitis, on the contrary, or of deep-seated caries of the vertebræ, the matter commonly gets under the iliac fascia, and follows the course of the iliac canal, being very deep and long before it appears externally; it either descends with the psoas and iliacus muscles to the thigh, or ascends to the flank and lumbar regions.

This, however, must be considered only as a general description; for as the matter may work its way through various tissues the abscess may be combined in different forms.

When abscess is seated underneath the fasciæ the case is always a dangerous one, because the matter almost always depends on some disease of the bones, or of the hip-joint; in the latter case the pus works its way from the joint into the synovial bursa placed between the articular capsule, the body of the pubis and iliac tendon; and as the bursa frequently communicates, either accidentally or naturally, with the cavity of the hip-joint, any infiltration of matter from the spine or pelvis may pass through it into the joint. Hence we often have disease of this latter part supervening on affections of the vertebræ, which terminate in abscess.

As I have already mentioned to you, the iliac canal leads from the iliac fossa to the upper part of the thigh; this circumstance explains the different degrees of depth at which abscesses form in the thigh in different persons. When the pus descends through the femoral canal, the abscess is subcutaneous; but when it passes under the iliac fascia, and through the inguinal canal, it must be deep-seated. In some cases the matter makes its way through the deep layer of the fascia iliaca, ascends towards the obturator foramen, and presents near the ischium; in other cases it works between the glutei muscles, triceps, and fascia lata, and points below the great trochanter. From this you may see how the pus may open at various points of the limb, and how we are able, in many instances, to ascertain the source of the matter from the place at which the abscess points.

From the pelvis the matter may pass into the groin through the obturator foramen, under Poupart's ligament, or even through a perforation of the cotyloid cavity; or the opposite of this may occur. In 1832 I saw a patient with abscess, which commenced underneath the fascia, behind the cotyloid cavity; the pus made its way through the inguinal and crural canals and the obturator foramen, then between the adductor and pectineus muscles, and winding round the neck of the femur, presented at the upper and outer part of the thigh. In another case the trajet of the abscess was the same, but it ascended as high as the edge of the tensor fasciæ femoris muscle."

We don't see any thing to notice in M. Velpeau's observations upon treatment.

---

### ROYAL BERKSHIRE HOSPITAL.

### Treacle and Water for Burns.*

Mr. Bulley used this with very good effect in a case of scald from melted pitch. In the first instance he employed a paste composed of equal parts of treacle and flour. We need not mention the particulars of the case, but content ourselves with the concluding remarks of Mr. Bulley.

"In recording the treatment adopted in the foregoing case, I do not wish to take any credit to myself for employing a remedy with the virtues of which, in such cases, surgeons have been long acquainted. Mr. Greenhow, of Newcastle, first introduced it into practice, using it as a defensative, for the purpose of preventing the access of air to the denuded parts. He did not, I believe, continue to use it throughout the whole progress of the case, but substituted for it other applications which the circumstances of the case might afterwards seem to require. In the commencement of the preceding case I used it, mixed with flour, for the same purpose of excluding air, but, finding it occasioned pain, and that I could not properly see what was going on underneath, although it had seemed to promote the growth of granulation, I determined to use equal parts of treacle and water, on rags, constantly applied as a lotion to the injured surface. The application of treacle in this manner has convinced me by the result of this, and other cases similarly treated, that it has some specific effect in expediting the cicatrisation of burns and scalds, however extensive they may be, and that it prevents, in a great degree, the unsightly puckering and contraction which too often interfere with the proper actions of joints involved in these accidents. I have, since that time, had several opportunities of testing its value as a remedy in these cases; and have, from what I have seen of its effects, adopted it in every case of the kind which has come under my care, both in hospital and private practice; and in each case it has seemed to have been instrumental in preventing, or at least diminishing, the chances of consecutive contraction."

---

### MIDDLESEX HOSPITAL.

### Dr. Watson on Cancer of the Stomach.†

From amongst some good remarks on cancer of the stomach we select one or two.

---

* Provincial Medical and Surgical Journal. Feb. 5, 1842.
† Medical Gazette, March 11, 1842.

*Occasional obscurity of Symptoms.*—" Not long since I saw, in consultation, an elderly clergyman, who complained of pains in his back, which were brought on or aggravated by certain movements of the body. His bowels were costive; and purgatives always relieved his pains. He was passing lithic acid gravel. The pains were felt in or near the renal region. Several years before he had suffered in a similar manner; and had then been cured by being cupped in the loins. What was the matter here? Was it lumbago? Was there a calculus in one of his kidneys? These were the best guesses that I could make. The eminent physician whom I met, and a surgeon of no less eminence, who had seen the patient previously, had not been able to attain any more exact diagnosis. Upon this gentleman's death, which occurred not long afterwards, his disorder was discovered to have been cancer of the stomach. Excepting slight sickness a day or two before he died, there had been no symptom to direct attention to that part."

*Pulsating Tumor occasioned by Scirrhus.*—A young woman came into the Middlesex Hospital, under one of Dr. W.'s colleagues, with a pulsating tumor in her epigastrium. It was thought, at first, to be an aneurism, and the case attracted, on that account, a good deal of notice. But the tumor subsided very much after free purgation. This led some to suppose that it was formed by accumulated fæces in the transverse colon. There was no sickness; nor indeed any one symptom referable to the stomach. She died. The tumor was cancerous; and in the stomach. Lying in front of the abdominal aorta, it had been lifted by its pulsations.

We were requested about a year ago, to meet a gentleman in consultation on a somewhat similar case. But the pulsating tumor was in the *left iliac* region. It was small and firm, and pulsated strongly. We gave it as our opinion that the case was either one of schirrhus of the omentum, or of the pylorus. It turned out to be the latter.

*General Laws with regard to the Symptoms.*—1st. That there is more suffering, *cæteris paribus*, when the cancerous disease is situated at, or very near, either extremity or orifice of the stomach, than when it occupies the intermediate parts; whether in the greater, or in the lesser curvature.

2nd. That when the cardia, and its immediate neighbourhood, is the part solely or principally diseased, the food and drink find a hindrance in passing into the stomach; but being once there, the distress is over. The symptoms are very like those of stricture of the œsophagus. The morsel reaches the bottom of that tube, and there causes uneasiness, till at length it is brought up again through the mouth, or passes gradually in the natural direction.

3rd. That when, on the other hand, the disease is limited to the pyloric end of the stomach, the food enters that bag readily enough, and remains there for a certain time; then uneasy sensations arise, and the imperfectly digested meal is apt to be rejected by vomiting.

---

### BRIDGEWATER INFIRMARY.

### Successful Amputation during the progress of Traumatic Gangrene.*

The case is related by Mr. Toogood, surgeon to the Infirmary.

---

* Prov. Med. and Surg. Journ. Feb. 26, 1842.

*Case.*—Charles Tuck, a farm servant, aged 24, received the contents of a common fowling-piece in the hand on Friday, the 4th of February, which passed up the flexor muscles and through the integuments of the fore-arm about three inches above the wrist. A neighbouring surgeon saw him soon after the accident, and directed cold applications and rest. On the following Monday he was admitted into the Bridgewater Infimary. There was a ragged wound at each opening, the limb was but little swollen, and the only unfavourable appearance was, that the nails looked rather dark coloured. There was very little constitutional disturbance. He was put to bed, a poultice applied over the whole limb, and the usual treatment directed. On the next day the aspect of the limb was the same; but as there was more swelling in the evening and some heat, leeches and fomentations were ordered. He slept perfectly well until five o'clock the following morning (Wednesday), when he was awoke by pain, which rapidly increased and became very severe. At seven o'clock the limb was found to be gangrenous to the elbow, and before his consent to its removal could be obtained, and the necessary preparation made, it extended so high up, as barely to leave room to amputate close to the shoulder joint. There was no line of demarcation, but I cannot, at this distance of time, recollect the precise point to which crepitus extended, but I am inclined to believe it was quite to the joint, as it was debated whether it would not be the safest plan to remove the bone from the socket. Very little blood was lost during the operation; the constitutional irritation subsided in a few hours; the patient became tranquil and soon recovered.

Every case of this kind is a valuable accession to the stock of practical knowledge.

---

### Tumors of Nerves.*

### 1. Dissection of a Tumor from the Sciatic Nerve.

The following case is taken from a Clinical Lecture on Tumors of Nerves, by M. Velpeau.

"I saw a case of ganglion which occupied the sciatic nerve; it was seated on the back of the thigh about four inches below the buttock; it had existed for several years, and acquired the size of a child's head. The tumor was extirpated in the following manner:—The patient was placed on his belly, with the legs separated, and supported by assistants, I made an incision six inches long from the ischium, dividing the subcutaneous fascia and the fascia lata; the tumor was thus exposed, drawn backwards, and dissected with caution from the edge of the biceps; it was now evident that the tumor was confounded with the sciatic nerve. The fear of gangrene or paralysis made me hesitate for a moment, and reflect whether it might not be possible to separate the tumor from the filaments of the nerve. I detached the circumference, and dissected the upper and lower parts of the nerve, as if I were making an anatomical preparation, and found that about one-third of its thickness was free, while the remainder was spread all over the bottom of the tumor. The patient bore this painful dissection with the greatest fortitude, and this gave me additional courage to proceed. I, therefore, dissected away each filament, pushed them aside, and at last succeeded in completely isolating the tumor. The cavity which remained was large enough to contain my two fists; I filled it with lint. During the first fortnight the patient complained of numbness and incapability of mov-

* Prov. Med. Journ. Feb. 26.

ing the parts about the foot and ankle, but these gradually disappeared, and she was completely cured in three months. M. Chelius and M. Roux have published similar cases: but in that mentioned by the latter surgeon the tumor was cancerous, and finally destroyed the patient."

### 2. PAINFUL NERVOUS TUMORS OF THE THORAX.

"The chest is frequently the seat of painful nervous tumors; I have often removed them from this part of the body. A lady suffered for many years from neuralgic pains in the right side of the chest. There was a small tumor, about the size and shape of an almond, between the tenth and eleventh ribs; and the pain seemed to originate from this point. In order to expose the tumor I had to divide the integuments to the extent of two inches, the subcutaneous fascia, some fibres of the dorsal and external oblique muscles, and the layer of tissue which covers the external intercostal muscles. When the tumor was raised up by a hook the patient experienced violent pain; it was easily removed, and complete recovery ensued in a short time. A year afterwards, however, I had to perform a similar operation on the same patient for another tumor, which appeared about an inch below and behind the first one. After this the patient had no relapse.

In 1836, there was a young girl at La Charité affected with a painful tumor in the same region, but on the opposite side of the body. In this case, although the ganglion was subcutaneous, I was unable to heal the wound by the first intention. I have seen a third case of painful ganglion in the same region, in a female; and am inclined to think that the friction caused by the petticoat or robe may have been an exciting cause of the disease."

---

### REPORT OF METROPOLITAN COMMISSIONERS OF LUNACY.*

The following is the Report of the Metropolitan Commissioners for the last five years.

Number of Houses under the Care and Inspection of the Commissioners.

| | |
|---|---|
| Pauper and private patients .. .. .. .. | 4 |
| Private patients only .. .. .. .. .. | 32 |
| Total .. .. .. .. | 36 |

| Expenses of Commission. | £. | s. | d. |
|---|---|---|---|
| Fees to two barristers .. .. .. .. .. | 2920 | 0 | 0 |
| Ditto to five physicians .. .. .. .. .. | 6498 | 0 | 0 |
| Salary of secretary .. .. .. .. .. | 2000 | 0 | 0 |
| Chaise hire, and other expenses .. .. .. .. | 2165 | 17 | 10 |
| Total .. .. .. .. | 13,583 | 17 | 10 |

Average cost of inspecting each house, £.388.

| Results. | |
|---|---|
| Licences refused .. .. .. .. .. . | 1 |
| Ditto, suspended .. .. .. .. .. .. | 0 |
| Ditto, revoked .. .. .. .. .. .. | 0 |
| Total .. .. .. .. .. | 1 |

---

* Lancet, March 19, 1842.

It further appears that, of the thirty-two houses for private patients—

5 are kept by resident medical proprietors.
4 situated respectively at Clapham, Upper Clapton, Chelsea, and Fulham, by physicians resident in London.
16 by resident proprietors, not being medical men.
7 by non-resident proprietors, not being medical men.
—
32

It also further appears, that of the counties under the joint jurisdiction of the commissioners and the quarter-sessions, 14 contain no licensed houses, and the remaining 26 contain 87 only, and these 87 houses do not include above 2500 patients of all ranks.

It further appears, also, that in 25 of these 26 counties, the returns of licences refused, suspended, and revoked, is *nil;* in the remaining county, viz. Dorset, the return is—Refused, 1; suspended, 0; revoked, 0.

Extract from the Annual Report of the Metropolitan Commissioners for 1838:—

"It appears from the returns already mentioned, that the total number of licensed asylums within England and Wales, exclusive of the metropolitan districts, is only about eighty, and that in a large majority of these the number of patients is extremely small; it further appears, that in several of the largest counties in England, and in the whole of Wales, not a single licensed asylum was to be found, a circumstance which the commissioners can hardly reconcile with facts that have otherwise come to their knowledge. It would seem to be a not unfair inference, that besides the houses for which licences have been taken out, in conformity with the Act of Parliament there must exist in different parts of the country other and unlicensed establishments in which persons are confined as insane patients (perhaps even without medical certificates) in contempt and defiance of the law. To what extent this practice prevails the commissioners have no means of forming an opinion, but it is one which may obviously lead to the most glaring and dangerous abuses, and which, therefore, strongly calls for a searching investigation and an effectual remedy."

Ditto, for 1841:—

With the view of rescuing persons, as far as may be, from unnecessary discomfort during their residence in these asylums, and in order to promote their liberation as soon as it can be effected with safety, the commissioners *have issued express direction (quere, when?)* that a sufficient number of keepers shall in all cases be employed, *so as to obviate the necessity of personal restraint, except in extreme cases;* and they have also endeavoured to establish some system of classification, in order that the convalescent patient may neither be confounded with the others, nor his recovery be retarded by associating with persons labouring under violent or confirmed mania.

It is satisfactory to add, that these endeavours have been seconded by the proprietors and superintendents of the three large houses, where classification is chiefly necessary. The commissioners feel bound to report, that those persons have *lately* bestowed much attention on this subject, and have, in fact, incurred considerable expense in carrying into practice the recommendations of the commissioners."

---

### Clinical Lecture on Varyx. By M. Velpeau. His Operation for Varicose Veins.*

A strong, sharp pin, with a large head and a waxed thread, are the only instruments that I use. The position of the patient is a matter of some importance.

---

* Prov. Med. Journal. March 19, 1842.

He must be placed in such a posture as will render the veins tumid and prominent. The trunk of the vein is now raised up with the fingers' end, the pin is passed below the ends of the nails and underneath the vein; it will be necessary to protect the finger with a thimble or a roll of linen, for the tissues under the varicose vein are often very hard and resistant. This simple process must be repeated with regard to every dilated vein; eight, ten, twelve, or fifteen pins may be required from the foot up to the knee, but, generally speaking, three or four are sufficient. When the veins are free and movable under the skin, it is easy to pass the pins under them; but, when they are applied closely to the bones, this is sometimes impossible; you must then pass a strong pin perpendicularly downwards, and then direct it obliquely under the trunk of the vein. The pins, being all placed, are fixed with threads; at first I applied the ligatures as we do for hare-lip, but this did not exercise sufficient pressure. I now twist the thread circularly round the pin, and draw it tight. The pins and ligatures are not removed before the sixth or twelfth day, when the tissues embraced between them have been destroyed. But even if the eschar be not detached at this period I remove them, because I feel confident that the vein is obliterated.

The introduction of the pin gives very little pain, but the constriction exercised by the ligature is excessively painful. Hence, you should commence with the highest pin, in order to cut off the nervous communication as much as possible. The effects of the operation are simple. The tissues embraced by the ligatures mortify and are separated, leaving a sore, which soon heals up. A hard chord forms above and below; this is the vein in process of obliteration. No dressing is required after this operation. You have merely to cut short the ends of the pins, to prevent them from pricking the patient. Unless the inflammation be severe the patient may go about; if it be excessive, then you confine him to bed, and have recourse to the antiphlogistic treatment. As soon as the eschars come away, the small sores which remain are treated as common abscesses or burns."

It seems that M. Velpeau has performed a great many such operations, with what success may be judged from the following confession.

"You may ask, however, if it always succeeds in establishing a radical cure. Our answer must be, No. The disease will not always yield. When one dilated vein is obliterated, three or four others soon appear, the superficial branches anastomose with the deep ones, and nothing is more difficult than to obstruct completely the circulation in a varicose limb. Hence the efficacy of every process or method that may be proposed for the radical cure of varix is extremely doubtful."

If this be the case why operate? We do not ourselves see the propriety of persevering in performing operations which, it is confessed, do not answer. For observe, that it is not a mere question of a few pins, more or less, in a man's leg, but one which concerns his very existence.

*Case.*—A man, 34 years of age, of good constitution and enjoying excellent health, was admitted into la Charité with an extensive varicose condition of the legs. The operation was performed in the usual way on the 4th of April. Several pins were passed under the veins, and the ligatures drawn tightly round them. The pain thus occasioned was very severe, and continued for several days; some slight inflammation set in around the pins. On the 10th two pins were removed, and on the 11th some more. Up to the 15th no untoward symptom appeared, but on the evening of that day the patient was seized with frequent shivering, nausea, and vomiting. On the morning of the 16th he was in the same state; the tongue was white; mouth dry; vomiting of green fluid; skin hot; headache and prostration; pulse quick; leg red and swollen. He was at once bled from the arm. On the 17th the vomiting ceased; there was delirium, slight stupor, pain of abdomen, and diarrhœa. The swelling of the leg extended up to the thigh; the pulse was small and frequent, and there were several livid spots on various parts of the body. On the 18th all the symptoms were aggravated, and the man sank on the following day.

On examining the body after death, the vessels were found full of very fluid blood, but there was no trace of pus in any of them. The vein which had been operated on was not obliterated.

Here was a man cut off in the prime of life by an operation, which *could not* have succeeded, for a mere inconvenience which a bandage *would* have remedied. We do not hesitate to tell M. Velpeau that operations of this description are carried on far too freely in France. He has many good points, and to set his face against the rage for operating that prevails in his own country would be the very best of them.

---

### Clinical Lecture of Sir B. Brodie on Abscesses *

Amongst other interesting and useful observations, Sir B. Brodie discusses the question whether matter once formed can be absorbed. He treats it thus:

Dr. Macartney states that he has seen the matter of a psoas abscess absorbed, and it has been said that the matter of a bubo has been also taken up. I recollect the case of a gentleman who had an abscess presenting over the ileum. I diagnosticated that there was dead bone; but in time the tumor, or abscess, or whatever else it was, entirely disappeared. A young man had scrofulous disease of the knee; it was packed up in splints and bandages, and in time all trace of the tumor disappeared. A young lady was affected with disease of the spine, and there was a hard solid tumor discoverable in the abdomen; this is now many years ago, and the last time I heard of her all trace of the tumor had disappeared, and she had quite recovered. Such facts as these would lead to the belief that the purulent matter poured out in these cases had been absorbed. I consider the point to be a very doubtful one on which to pass a certain decision. A man was in this hospital with lumbar abscess; in course of time it disappeared, and re-appeared again in its original situation. I have opened many suppurating buboes, but without meeting with any pus; and it frequently happens in these cases, that the soft fluctuation felt under the skin arises from a collection of serum between the gland and the skin, and nothing more. A girl had a tumor over the sternum, which inflamed; I pierced it with a lancet, and a large collection of serum escaped; this got quite well; by and by another tumor formed in the same place of a similar kind. I did not open this one, and in process of time it disappeared spontaneously; and, if I had not opened the first tumor, I dare say that it would have been absorbed.

---

### NORTHERN HOSPITAL, LIVERPOOL.

### Successful Amputation during Spreading Traumatic Gangrene.†

A few pages back we have noticed a successful case of amputation during the progress of traumatic gangrene, by Mr. Toogood, of Bridgewater. Here is another, evidence enough of the propriety of the practice. Our readers will not fail to observe that both are instances of gangrene of the *upper* extremity.

---

* Prov. Med. Journ. March 5, 1842. † Med. Gaz. Feb. 25.

*Case.*—David Wright, æt. 23, admitted Sept. 12, 1841, was brought to the hospital early in the morning in a state of intoxication, having been engaged in a street fight. The left arm presents the following appearances : there is a wound at the inner side of the elbow joint three inches in length, extending obliquely over the inner condyle of the left humerus, which, with the trochlea surface, lies exposed in the wound, but does not protrude; the ligamentous and tendinous structures at the inner side of the joint are completely divided, and two small portions of bone, detached from the interior condyle, can be felt in the wound; the olecranon process projects backwards remarkably, and on further examination it appears that the joint has sustained a dislocation of both bones backwards. On introducing the finger into the wound, and grasping the parts in front of it, no pulsation of the brachial artery can be perceived; the sensibility of the hand and fingers is unimpaired : considerable venous hemorrhage seems to have taken place, but the bleeding has now nearly ceased. After removing the loose fragments of bone, and reducing the dislocation (which was very easily effected by slight extension), the edges of the wound were brought into apposition by strips of adhesive plaster, over which a piece of lint dipped in blood was applied : the fore-arm was brought to a right angle with the arm, and the joint kept perfectly quiet by being laid on a tin splint. On the morning of the 15th, a gangrenous spot was observed at the inner side of the fore-arm. On the 16th, the gangrene had considerably extended, involving nearly the whole of the fore-arm, and extending rapidly to the arm; no appearance of a line of demarcation could be observed; the countenance had become anxious, features sharpened, pulse 120, and weak. A consultation was then called, at which it was resolved to perform immediate amputation of the member, which was accordingly done four inches below the shoulder. The operation was performed by the circular method, but presented no point of interest; the stump went on favourably, his general appearance progressively improved, and on the 19th Oct. he was discharged.

---

### DIAGNOSIS OF ASCITES AND OVARIAN DROPSY BY PERCUSSION.

Dr. Watson, in his lectures, points out the manner of distinguishing these affections by percussion.

In true ascites the relative place of the liquid and of the intestines is determined by the posture of the patient. The bowels, which always contain some gas, float to the upper part of the liquid, and there give out (when the finger, as a pleximeter, is applied to the corresponding surface, and struck) their peculiar resonance. Mediate percussion will thus follow the gravitating fluid, and discover always a dull sound in the lowermost and a hollow sound in the uppermost part of the abdomen.

But it is not so in ovarian dropsy. The cyst, in a diseased and enlarging ovary, rises in front of the intestines, which, being tied down by the mesentery, cannot embrace the tumor so as to reach its anterior aspect, but are in fact pressed back by it towards the spine. Hence, if there be any resonance produced by percussion, it is in one, or the other, or in both, of the flanks ; and the umbilical region yields a dull sound whatever the position of the patient may be. The same is true of the enlarging womb in pregnancy.

This simple expedient, then, is quite decisive. In ascites the epigastric or umbilical region is tympanitic on percussion; in ovarian dropsy it is dull. To be quite sure it is well to make the patient assume different postures in succession. If the person affected with ascites turns upon her side, the uppermost flank will

---

* Med. Gaz. Feb. 25.

become resonant; the umbilical region dull: whereas in ovarian dropsy, the sounds remain severally where they were under every change of position.

Dr. Watson alludes to three exceptional cases.

1. The distention, in true ascites, may be *so* great, that the mesentery shall not be broad enough to allow the buoyant intestines to reach the surface, when the patient is supine. Dr. W. has seen such a case.

2. The intestines may be tied down, and so prevented from ascending, by their specific lightness, to the upper part of the surrounding liquid. And this may happen, either in consequence of the adhesion of the various coils of the intestines to each other, and to the parts behind them; which is not an uncommon occurrence:—or the intestines, though unadherent, may be swathed, as it were, and bandaged down, by a thickened and diseased omentum.

3. Dr. W. once knew an ovarian cyst to exist, when the umbilical region was tympanitic under percussion. The case furnished just that kind of exception which serves to prove a rule. This was also a hospital patient. Her *history* was the history of ovarian dropsy. Some time previously she had discovered a small tumor in one of the iliac regions. It increased without much disturbance of her general health, until it became very inconvenient from its bulk. She was then tapped in one of the Borough hospitals: and she stated distinctly that it was not a clear watery fluid that was evacuated; but a glutinous, mixed, and grumous matter: such as belongs to ovarian disease. No doubt could be entertained that the enlargement of the abdomen resulted from disease of that kind. Yet the umbilical region, when percussed, always rendered a hollow sound. Upon the death of the patient the mystery was solved. Air hissed forth from the opening made by the scalpel through the abdominal parietes: and the source of it being traced, an ovarian cyst, of considerable magnitude, was found adhering to the peritoneum in front of the belly, and containing no liquid, but some yellowish shreds only; the remains, apparently, of some smaller included cysts. This ovarian bag had been filled with air, and had given occasion to the equivocal sounds.

Dr. Watson alludes to the possibility of confounding a much distended bladder with ascites. John Hunter once made this mistake, and operated.

---

### ST. MARYLEBONE INFIRMARY.

### Diuretics in Phthisis, Chronic Catarrh, &c.*

Dr. Clendinning, in some clinical lectures that he has been delivering, has been directing attention to the use of diuretics in the above-mentioned affections. His reasoning is, at the very least, ingenious. "Now," says he, "the use of these diuretics in cases where what are called expectorants are commonly chosen, seems worthy of trial in many cases of chronic pulmonary catarrh, simple or complicated. There is no use, I think, in stimulating action in the exhalent surfaces or secreting vessels of the air-passages by means of expectorants, except in the acute or actively inflammatory stages and states of catarrhs of the lungs. When the disease has become indolent, chronic, or passive, the best treatment is that which is most analogous to the proper management of other habitual fluxes or excessive discharges; namely, first, by direct repression of the disease by inhalation of appropriate vapours or gases, or circuitously by means of astringents, such as lead, alum, zinc, or copper, by the stomach, &c.; and, secondly, by derivatives which cause determinations of blood, and of nervous action to

* Lancet, March, 12.

other organs and tissues, and therefore away from the surface of the air-tubes; such are emetics, purgatives, blisters, warm-baths, &c. This view is confirmed by the partiality of some of the first authorities of the profession to several drugs which are unquestionably diuretics, and are also supposed to be possessed of expectorant powers; I mean squill, terebinthinates, particularly copaiba and other balsams, lobelia, nitrous ether, &c. &c. The occasional efficacy of each of those in chronic catarrh is unquestionable, and the diuretic property of each of them is quite certain, but their expectorant power in some measure, if not altogether hypothetical. The view I am now explaining is further confirmed by the fact, that in chronic catarrhs, antimony, and tartar-emetic particularly, are rarely employed as expectorants merely, while ipecacuan is less frequently used very much and less successfully, I am satisfied, than in acute bronchitis; yet none can doubt the power of either of those over acute inflammations of the pulmonary surfaces or substance."

---

### KING'S COLLEGE HOSPITAL.

### MR. FERGUSSON ON THE CIRCULAR AND FLAP OPERATIONS.*

We all know how incontinently some surgeons laud the flap amputation, and how they affect to look down on the circular, while their pupils, taught *jurare in verba magistri*, follow open-mouthed and in full cry upon the same scent. But sober-minded people are not caught by clamour, and quietly judge for themselves. Such a person is Mr. Fergusson, acknowledged to be a highly dexterous operator. We quote from one of his Clinical Lectures.

Mr. Fergusson said that he had removed the limb by what was usually called the double-flap operation; and his chief reason for selecting this method was, that he was more in the habit of performing the flap operation than the circular. Many surgeons were in the constant practice of performing the latter method in this part of the body as well as in others. Some had imagined that in consequence of the flat shape of the fore-arm, and the soft parts being chiefly in front and behind, it was particularly advisable to make the stump of flaps. He thought the choice of methods of less moment than many appeared to do. He thought that if a circular operation were properly performed, as good a stump might be produced as by the most adroit performance of the flap. Though he considered himself an advocate of the flap operation in general, he did not, in consequence, condemn the circular. He was of opinion, that the question of superiority had never in this country been fairly tested, for the advocates on either side were in the custom of confining themselves to the performance of only one kind of operation, and therefore, from their own experience, could not give an unprejudiced verdict. He did not admit that a person who invariably amputated with a flap could give such a decision, nor could it be expected from him who only followed the circular method. He should deem that opinion to be most entitled to consideration, which was given by a party who had operated an equal number of times, say fifty of each, by the two methods.

---

* Lancet, March 12, 1842.

